# Rising Star Early-Career Faculty Award Lecture and Cyber-Pedagogy Panel Discussion

**DOI:** 10.1093/geroni/igab046.1051

**Published:** 2021-12-17

**Authors:** Kara Dassel

## Abstract

The Rising Star Early-Career Faculty Award lecture will feature an address by 2021 recipient Candace S. Brown, PhD, MA, MEd, of the University of North Carolina, Charlotte. The Rising Star Early-Career Faculty Award acknowledges new faculty whose teaching and leadership stand out as influential and innovative. This event will also feature a panel discussion led by the AGHE Awards Review Panel titled, "Cyber-Pedagogy to the Rescue: Creating Effective Online Programming for Students and Trainees During the Pandemic.”

